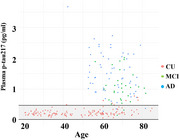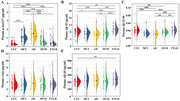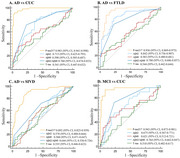# Diagnostic and discriminative accuracy of plasma phosphorylated tau 217 for symptomatic Alzheimer's disease in a Chinese cohort

**DOI:** 10.1002/alz70856_099222

**Published:** 2025-12-24

**Authors:** Ping Che, Nan Zhang

**Affiliations:** ^1^ Tianjin Medical University General Hospital, Jin Tian, Jin Tian, China; ^2^ Department of Neurology, Tianjin Medical University General Hospital, Jin Tian, Jin Tian, China

## Abstract

**Background:**

Plasma phosphorylated tau at threonine 217 (*p*‐tau217) measured with an ultrasensitive immunoassay method has been demonstrated to be an optimal biomarker for Alzheimer's disease (AD). This study aimed to establish the reference interval of plasma *p*‐tau217 in Chinese individuals and evaluate its diagnostic value in symptomatic AD.

**Method:**

We recruited 150 cognitively unimpaired (CU) individuals, 60 patients with AD dementia, 30 patients with mild cognitive impairment (MCI) due to AD, 40 patients with frontotemporal lobar degeneration (FTLD), and 70 patients with subcortical ischemic vascular dementia (SIVD). The concentrations of plasma *p*‐tau217, total tau, amyloid‐beta (Aβ)42 and Aβ40 were measured with a single‐molecule array.

**Result:**

Plasma *p*‐tau217 outperformed other biomarkers in discriminating AD patients from CU controls, FTLD patients, and SIVD patients (AUC = 0.983, 0.936, 0.892), and discriminating MCI patients from CU controls (AUC = 0.943). The plasma *p*‐tau217 level was negatively correlated with memory in patients with symptomatic AD.

**Conclusion:**

Plasma *p*‐tau217 demonstrated exceptional diagnostic accuracy for AD even at early stages in the Chinese population.